# Intermediate strain rate constitutive modeling of gradient rolled vanadium-microalloyed pearlitic steel for bridge cables

**DOI:** 10.1016/j.isci.2026.116319

**Published:** 2026-06-10

**Authors:** Sheng Huang, Zhiying Li, Hui Yang, Zeyun Zeng, Yingjie Shi, Changrong Li

**Affiliations:** 1 College of Materials and Metallurgy, Guizhou University, Guiyang 550025, China; 2 Guizhou Provincial Key Laboratory of Metallurgical Engineering and Process Energy Saving, Guiyang 550025, China

**Keywords:** Applied sciences, Materials science, Alloys

## Abstract

Ensuring the structural integrity of bridge cables under dynamic loads requires materials with stable strain-rate-dependent mechanical properties. This study investigates the rate-dependent tensile behavior and constitutive modeling of vanadium-microalloyed pearlitic steel processed via gradient rolling (10%–40% reduction) at intermediate strain rates (10^−4^ to 1.3 s^−1^). Experimental results show that synergistic strengthening from vanadium precipitation and gradient rolling achieves yield strengths exceeding 700 MPa and ultimate tensile strengths over 1,299 MPa. While gradient rolling maintains work-hardening capacity, elevated strain rates induce non-linear ductility loss due to a transition from dimple rupture to quasi-cleavage failure. To address these rate-dependent responses, a modified Johnson-Cook model incorporating a cubic polynomial coupling term was developed. This model provides accurate predictions for intermediate strain-rate stress-strain behaviors, offering a framework for the safety assessment and design of advanced bridge cable systems.

## Highlights

• Gradient rolling and V-microalloying optimize the strength-ductility balance
• Nanoscale V(C,N) precipitates induce quasi-cleavage at elevated strain rates
• A cubic polynomial-modified J-C model accurately predicts dynamic flow stress

## Graphical abstract

**Figure:**
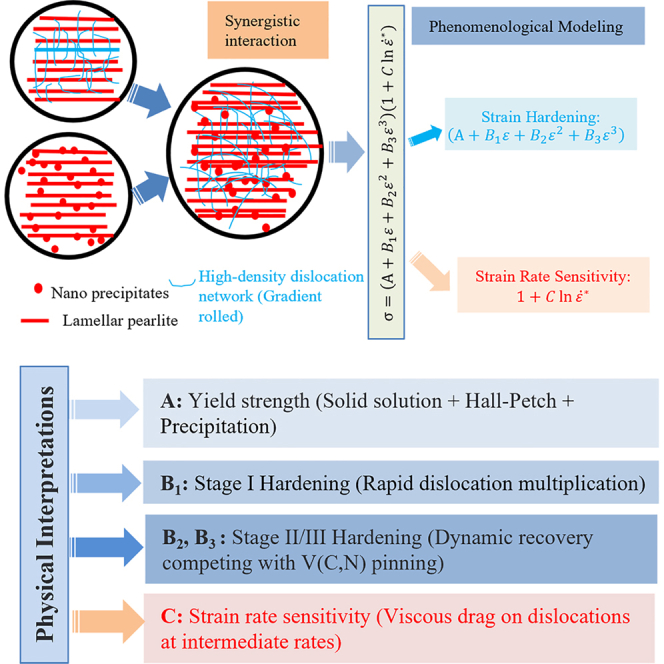

## Introduction

High-carbon pearlitic steel, characterized by its distinctive lamellar ferrite/cementite structure, is the preferred material for critical bridge cable applications, such as high-strength steel wires and prestressed strands. While it offers an excellent balance of strength and toughness under static loading, long-span bridges are inevitably exposed to multi-source dynamic loads during service, including seismic excitations, wind-induced vibrations, and vehicle impacts. With the growing demand for lightweight solutions in ultra-long-span bridge construction, the industry is pushing for next-generation cable steels with ultrahigh strength (e.g., increasing from 1,860 to 1,960 MPa to reduce cable cross-sectional area by 5.1%).^1^ However, this strength enhancement often introduces risks associated with reduced ductility and altered dynamic response sensitivity. Therefore, understanding the strain-rate dependent behavior and establishing an accurate dynamic constitutive model is a prerequisite for the nonlinear dynamic analysis and safety assessment of bridge structures. Consequently, achieving a breakthrough in material design through optimized microalloying and processing, while simultaneously characterizing its dynamic mechanical constitutive relation, has become a critical focus in civil and mechanical engineering.

Vanadium microalloying has proven effective in balancing the strength-toughness trade-off in high-carbon steels. Through its ability to control precipitation,^2^^,^^3^^,^^4^ vanadium enables a dual strengthening mechanism: (1) Zener pinning by VC nanoprecipitates at austenite grain boundaries, which effectively inhibits grain coarsening^5^^,^^6^; and (2) homogeneous dispersion of these nanoprecipitates within the matrix, which impedes dislocation motion.^7^^,^^8^^,^^9^^,^^10^ For bridge cable applications, where maintaining ductility is as critical as achieving high strength, the precipitation kinetics of vanadium play a decisive role. Gradient rolling, known for inducing non-uniform strain fields, has proven effective in grain refinement and strength enhancement in medium- and low-carbon steels. However, the microstructural response of high-carbon pearlitic steel—characterized by its lamellar structure—to such gradient strain fields remains unclear. This knowledge gap is particularly pronounced when vanadium microalloying is introduced, as the coupling effect between strain-induced precipitation and the gradient strain field is rarely reported.

Notably, recent studies have demonstrated that under dynamic deformation conditions, nanoscale precipitates not only strengthen the matrix via dislocation pinning but can also activate ultrahigh strength and ductility in ultrafine-grained steels.^11^^,^^12^^,^^13^ Addressing this gap is crucial for advancing the application of this processing route in high-performance bridge cable steels. Specifically, how the synergistic interaction between the gradient dislocation network and nanoscale V(C,N) precipitates influences the intermediate strain-rate mechanical response remains a critical knowledge gap.

Gradient rolling, which imposes a non-uniform strain field, enables multi-scale microstructural regulation. This technique has demonstrated significant advantages in medium- and low-carbon steels. For example, in AISI 4140 steel, gradient rolling induces a dynamic ferrite phase transformation, resulting in an ultrafine-grained structure and an increase in yield strength to 1,570 MPa.^14^ Similarly, in high-manganese steels, it modulates dislocation proliferation and delays dynamic strain aging.^15^ In contrast, high-carbon pearlitic steels—constrained by their lamellar cementite structure and low driving force for phase transformation—exhibit a fundamentally different response to gradient deformation. It is hypothesized that in vanadium-microalloyed high-carbon steels, strain-induced precipitation may spatially couple with the gradient strain field, giving rise to a unique dislocation network. Elucidating this process-structure relationship is essential for optimizing the manufacturing of next-generation bridge cables.

Gradient rolling inherently creates a high and heterogeneous initial dislocation density. Under intermediate strain rates, the mobile dislocations generated during testing interact abruptly with this pre-existing dense dislocation forest and the V-precipitates. This severely restricts the thermal activation volume for dislocation glide, making the material’s flow stress more sensitive to strain rate variations compared to ordinarily rolled materials. The Johnson-Cook (J-C) model and Cowper-Symonds (C-S) model are the most widely adopted frameworks due to their mathematical robustness and ease of implementation in finite element analysis (FEA).^16^^,^^17^ In the field of civil and mechanical engineering, these models have been extensively applied to predict the dynamic response of structural steels, with researchers such as Chen et al.^18^^,^^19^^,^^20^ and Liu and Soares^21^ significantly improving prediction accuracy through refined parameter determination. Recent investigations have specifically focused on the constitutive modeling of high-performance constructional steels under dynamic loading. For instance, Li et al.^22^ systematically investigated the high-strain-rate mechanical behaviors of Q390D steel via quasi-static tensile tests and split Hopkinson pressure bar (SHPB) experiments, verifying that a modified J-C model outperforms the original in predicting plastic flow stress. Similarly, Wang et al.^23^ calibrated the J-C model parameters for Q355B steel with different plate thicknesses through parameter inversion, providing reliable constitutive support for structural dynamic simulation. Building on these engineering applications, the research focus has concurrently shifted toward enhancing model fidelity by incorporating microstructural evolution under high strain rates^24^ and optimizing hardening terms to capture non-linear plasticity in advanced alloys.^25^

However, standard constitutive formulations often exhibit limitations when applied to advanced materials with complex hardening behaviors. While the standard J-C model is widely integrated into commercial finite element (FE) codes due to its mathematical simplicity, its classical power-law hardening term fundamentally fails to capture the multi-stage strain hardening behaviors induced by heterogeneous microstructures. For instance, conventional J-C models frequently fail to capture the non-linear strain-rate sensitivity of high-strength steels, as noted by Yang^26^^,^^27^ and Tang et al.^28^ in their studies on S690 and other high-strength grades. To address these limitations, alternative model forms have been extensively explored in recent years. On one hand, physically based models, such as the Zerilli-Armstrong (ZA) model or dislocation-density-based models,^29^^,^^30^^,^^31^ offer deep microstructural insights but require extensive parameter calibration and high computational costs. On the other hand, phenomenological models modified with exponential or polynomial terms provide a pragmatic balance between predictive accuracy and engineering applicability.^32^^,^^33^ For vanadium-microalloyed high-carbon pearlitic steel, specifically under the coupled effects of gradient rolling and dynamic loading, the strain hardening behavior becomes highly non-linear due to the synergistic interaction between dense dislocation forests and nanoscale V(C,N) precipitates. Consequently, existing models remain inadequate, and developing a modified constitutive formulation that accurately couples this complex strain hardening with rate effects is a critical frontier for ensuring the reliability of numerical simulations.

While the quasi-static mechanical properties of high-carbon steels have been systematically characterized,^34^^,^^35^^,^^36^^,^^37^^,^^38^^,^^39^^,^^40^^,^^41^ a significant gap persists regarding their dynamic behavior and constitutive modeling, particularly for bridge cable applications. The specific influence of strain rate on the failure mechanism of gradient-rolled, vanadium-microalloyed steel remains largely unexplored. This study aims to bridge this gap by systematically investigating the coupled effects of gradient rolling (10%–40% reduction) and dynamic loading (10^−4^–1.3 s^−1^). The specific objectives are to: (1) quantify the dynamic mechanical enhancement; (2) elucidate the transition in fracture mechanisms; and (3) develop a modified J-C model with a cubic polynomial coupling term. This work provides both experimental data and theoretical tools necessary for the impact resistance design and safety assessment of long-span bridges.

## Results

**Figure 1:**
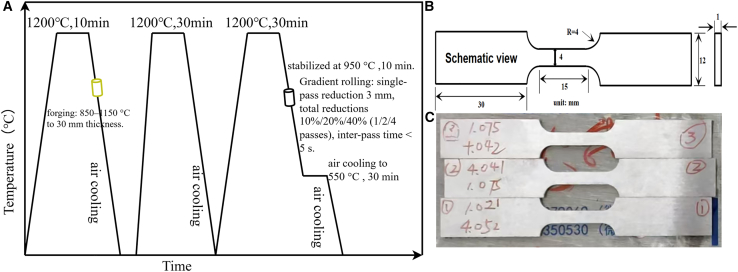
Sample processing process flowchart and tensile sample diagram (A) Processing technology curve. (B) Schematic diagram. (C) Actual sample image.

The chemical compositions of the investigated high-carbon pearlitic steels (steel A and steel B) are detailed in Table 1. The specific thermo-mechanical processing route, including the gradient rolling reductions, and the specimen geometry used for intermediate strain-rate tensile testing are illustrated in Figure 1 (see STAR Methods for full details).

**Table 1:** Chemical composition of experimental steel (wt. %)

Steel	C	Si	Mn	Cr	P	S	V	N	Fe
A	0.82	0.71	0.47	0.50	≤0.01	≤0.01	0	0.00032	Bal.
B	0.82	0.71	0.47	0.50	≤0.01	≤0.01	0.086	0.00370	Bal.

### Engineering stress-strain behavior

**Figure 2:**
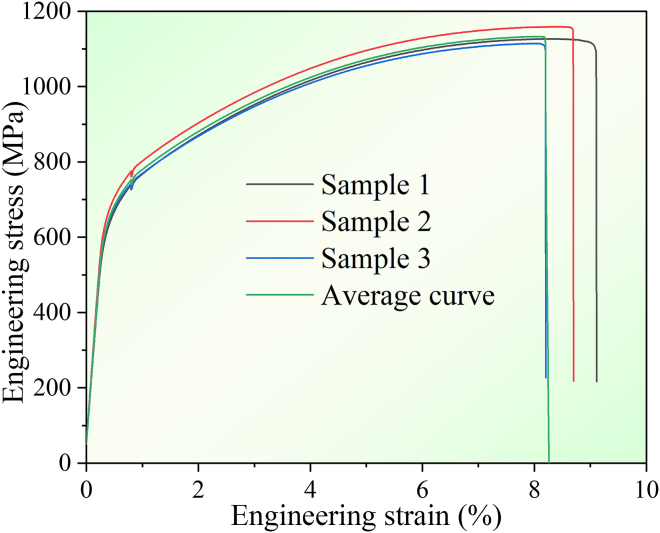
B-R40 engineering stress-strain curve: strain rate of 1 × 10^−4^ s^−1^

Figure 2 illustrates the engineering stress-strain reproducibility for the vanadium-microalloyed steel (specimen B-R40) under quasi-static loading conditions (1 × 10^−4^ s^−1^). To ensure statistical reliability and mitigate the experimental scatter inherent to the localized deformation of high-strength pearlitic steels, three independent tests were conducted for each condition. An average response curve was generated, and the experimental trial exhibiting the minimal deviation from this average was selected as the representative curve for subsequent constitutive analysis.

**Figure 3:**
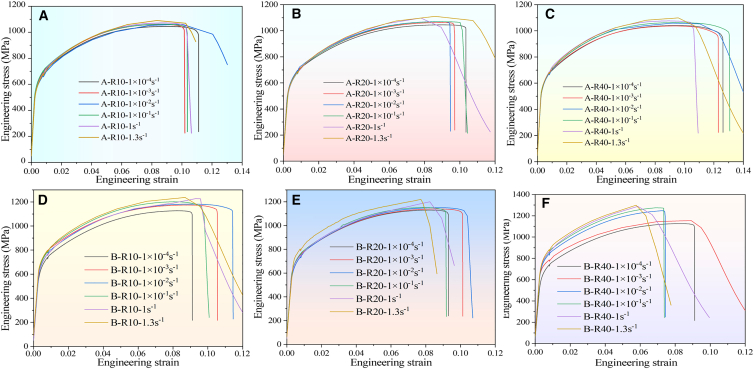
Engineering stress-strain curve (A) A-R10. (B) A-R20. (C) A-R40. (D) B-R10. (E) B-R20. (F) B-R40.

The representative engineering stress-strain curves for steel A and steel B under varying rolling reductions (10%, 20%, and 40%) and strain rates are presented in Figure 3. A distinctive feature across all specimens is the continuous yielding behavior, characterized by the absence of a Lüders plateau. This is attributed to the high density of mobile dislocations introduced by the gradient rolling process, which unpins the Cottrell atmospheres. Given this behavior, the yield strength was determined using the 0.2% offset method in accordance with ASTM E8/E8M standards. A clear positive strain rate sensitivity is observed: as the strain rate increases from 10^−4^ to 1.3 s^−1^, the flow stress shifts upward significantly, enhancing the load-bearing capacity under dynamic conditions.

**Figure 4:**
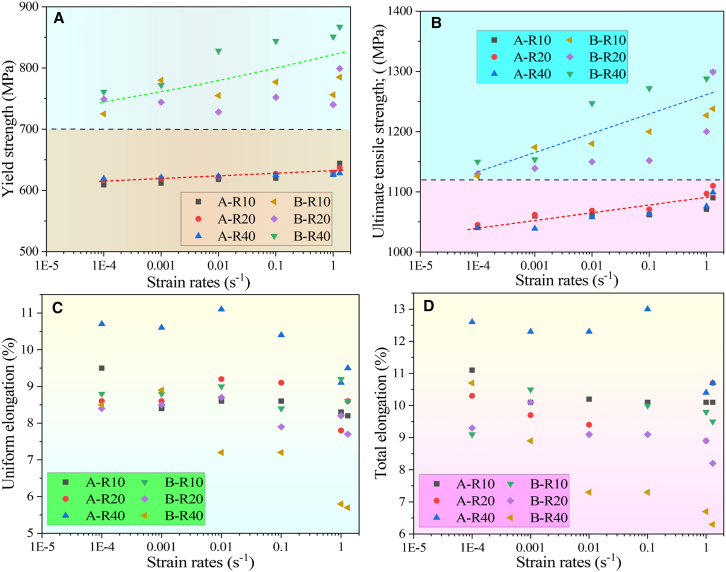
Evolution of mechanical properties as a function of strain rate and rolling reduction for steel A and steel B (A) Yield strength. (B) Ultimate tensile strength. (C) Uniform elongation. (D) Total elongation.

The mechanical properties of steel A and steel B under varying rolling reductions and strain rates, covering yield strength, ultimate tensile strength, uniform elongation, and total elongation, are presented in Figure 4 to facilitate the analysis of their interrelationships.

Figures 3, 4A, and 4B reveal a hierarchical strengthening mechanism. First, strain rate hardening is evident, with yield strength increasing logarithmically with strain rate. Second, vanadium microalloying provides a substantial baseline strength increment; steel B consistently exhibits 15%–25% higher yield strength than steel A under identical conditions, surpassing 700 MPa. Third, gradient rolling induces sustained work hardening rather than a simple monotonic surge in strength. For instance, at quasi-static conditions, increasing the rolling reduction from 10% to 40% in steel B elevates the ultimate tensile strength modestly from 1,126 to 1,150 MPa. As highlighted in recent literature,^42^^,^^43^ the true value of such microstructural design lies not in a drastic strength spike, but in effectively optimizing the strength-ductility balance. By sustaining structural integrity without suffering a severe drop in plastic stability, the material maintains its exceptional load-bearing capacity. Notably, at the highest tested strain rate (1.3 s^−1^), this value reaches a peak of 1,299 MPa, demonstrating the material’s robust capacity to resist intermediate strain rate loads (such as wind-induced vibrations and traffic excitations).

**Figure 5:**
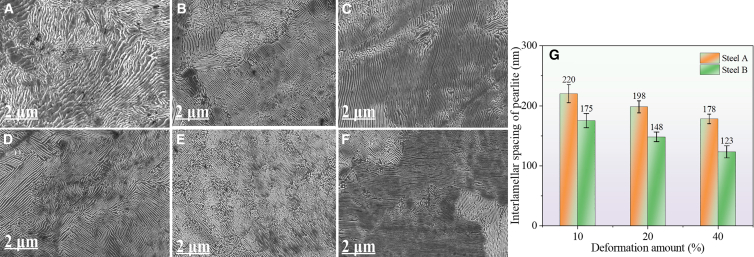
SEM morphology (A) A-R10. (B) A-R20. (C) A-R40. (D) B-R10. (E) B-R20. (F) B-R40. (G) Interlamellar spacing of pearlite (scale bars, 2 μm).

The mechanical response reveals a synergistic interaction between gradient rolling deformation and vanadium microalloying. As the rolling reduction increases from 10% to 40%, a significant upward trend in both yield and tensile strengths is observed (Figures 4A and 4B). This strengthening is primarily attributed to grain fragmentation and the proliferation of dislocation networks, which effectively impede dislocation motion.^44^ This macroscopic strengthening is fundamentally governed by microstructural evolution. To quantitatively substantiate this mechanism, the pearlite interlamellar spacing (*λ*) under varying gradient rolling reductions was statistically evaluated via high-magnification SEM imaging (with over 100 measurements per condition). As shown in Figure 5.

The analysis reveals a pronounced geometric refinement: increasing the rolling reduction from 10% to 40% refines the average interlamellar spacing from approximately 220 nm to 178 ± 8 nm in steel A, and from 175 nm down to 123 nm in the vanadium-microalloyed steel B. This quantifiable refinement severely restricts the dislocation mean free path, thereby elevating the macroscopic yield and tensile strengths in strict accordance with the boundary strengthening mechanism (Hall-Petch relationship, *σ*_*y*_∝ *λ*^−1/2^).

In steel B, this effect is further amplified by the precipitation of fine vanadium carbonitrides (confirmed in Figure 10), which contributes to strength via Orowan looping and grain boundary pinning mechanisms.^45^^,^^46^ However, this strength enhancement comes at the thermodynamic cost of ductility. As shown in Figures 4C and 4D, both uniform and total elongation exhibit a non-linear reduction with increasing rolling reduction and strain rate. Specifically, the coupling of excessive rolling reduction (40%) and intermediate strain rates (1.3 s^−1^) leads to a noticeable drop in ductility. This phenomenon is physically governed by dislocation saturation and the exhaustion of work-hardening capacity (limited capacity for further plastic flow), rather than macroscopic defects. Crucially for structural integrity, although steel B exhibits reduced ductility compared to the reference steel A due to the dual influence of solid solution and precipitation hardening, it maintains a total elongation exceeding 6% even under the most severe loading conditions. This residual ductility indicates that the vanadium-microalloyed steel retains sufficient energy absorption capacity to prevent catastrophic brittle failure, thereby meeting the safety requirements for bridge cables under seismic loading frequencies.

Regarding the absolute strength level, comparison with commercial standards is essential. It is important to note that commercial bridge cable wires (typically 1,860 or 1,960 MPa) are achieved through multi-pass cold drawing of sorbitized wire rods. The material investigated in this study represents the hot/warm rolled precursor state. Achieving a tensile strength of ∼1,300 MPa with >6% ductility in the as-rolled state (steel B-R40) significantly exceeds the performance of conventional pearlitic wire rods (1,000–1,100 MPa). However, while this superior precursor strength is promising, simple linear extrapolation to ultra-high strengths (e.g., >2,200 MPa) via subsequent cold drawing must be treated with caution. Because the gradient rolling process has already introduced a high density of dislocations and precipitates, a portion of the material’s plasticity has been intrinsically consumed. Therefore, whether subsequent cold drawing can reach 2,200 MPa while maintaining the requisite torsional ductility remains a critical challenge. Future industrial processing must carefully balance these competing mechanisms, as similarly emphasized in recent strategies for boosting strength without sacrificing ductility.^47^

### True stress-strain behavior and work hardening characteristics

**Figure 6:**
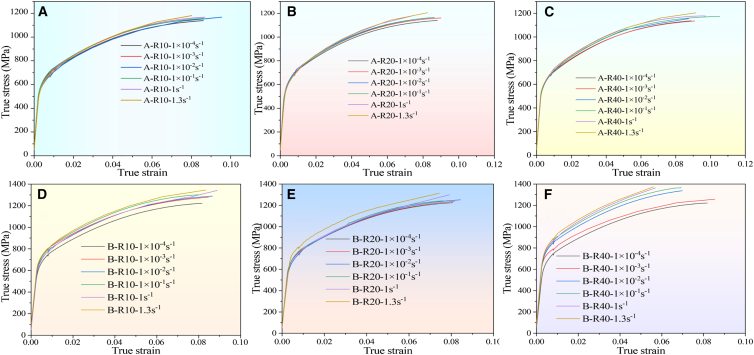
True stress-strain curves (A) A-R10. (B) A-R20. (C) A-R40. (D) B-R10. (E) B-R20. (F) B-R40.

The engineering stress-strain data were converted to true stress (*σ*) and true strain (*ε*) relationships based on the instantaneous cross-sectional area assumption (volume conservation). Figure 6 presents the resulting true stress-strain curves across the tested strain rate spectrum (10^−4^ to 1.3 s^−1^).

It is critical to note that the curves presented are intentionally truncated at the point of plastic instability (corresponding to the ultimate tensile strength). By restricting the analysis to the uniform deformation stage, the validity of the volume conservation assumption is strictly maintained. This approach effectively circumvents the complex stress triaxiality induced by necking, thereby eliminating the need for empirical post-necking corrections such as those proposed by Bridgman.^48^ Consequently, a physically rigorous dataset is ensured for the subsequent constitutive modeling. Compared to the engineering curves, the derived true stress exhibits a steeper strain hardening response, accurately reflecting the material’s increasing resistance to plastic flow. Furthermore, as evident in Figure 6, the flow stress demonstrates a distinct positive strain rate dependency, where elevated strain rates amplify the flow stress through the suppression of thermal activation for dislocation motion.^49^

**Figure 7:**
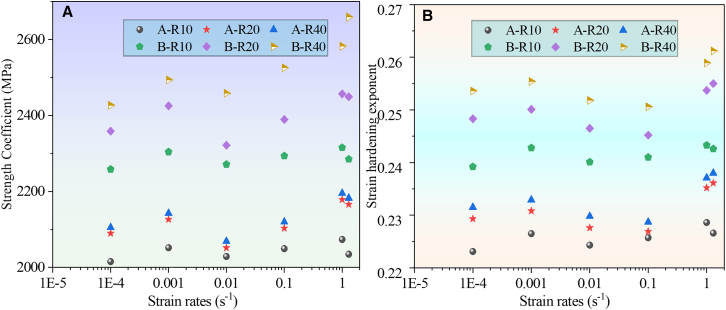
Dependence of Hollomon constitutive parameters on strain rate and rolling reduction for steel A and steel B (A) Strength coefficient (*K*). (B) Strain hardening exponent (*n*).

To quantitatively evaluate the strain hardening capacity, the plastic flow behavior in the uniform deformation stage was analyzed using the Hollomon power-law relationship: *σ* = *K·ε*^*n*^, where *K* is the strength coefficient and *n* is the strain hardening exponent. The regression analysis yields high coefficients of determination (R^2^ > 0.99) confirming the validity of the power-law description for the uniform plastic flow of these pearlitic steels. The evolution of *K* and *n* with strain rate and rolling reduction is visualized in Figure 7, revealing three critical insights into the process-structure-property relationship.

As depicted in Figure 7A, the strength coefficient (*K*) exhibits a clear stratification based on material composition and processing history. Steel B consistently exhibits *K*-values approximately 200–400 MPa higher than those of steel A under identical conditions. For instance, at a strain rate of 1.3 s^−1^, the *K*-value peaks at 2,659 MPa for B-R40, compared to 2,182 MPa for A-R40. This substantial increment confirms the efficacy of precipitation strengthening induced by vanadium carbonitrides, which acts synergistically with the dislocation multiplication caused by gradient rolling to enhance the flow stress level. Furthermore, the positive slope of the K-curves with respect to strain rate confirms the material’s rate-dependent strengthening mechanism, attributed to the viscous drag on dislocation motion.

Typically, severe cold working exhausts a material’s work-hardening capacity, leading to a drastic drop in the *n*-value. However, a counter-intuitive yet advantageous phenomenon is observed in Figure 7B. As the rolling reduction increases from 10% to 40%, the *n*-values do not degrade; instead, they maintain stability or show a slight increment (e.g., rising from 0.24 to 0.26 for steel B). To elucidate this mechanism, it is essential to consider the microstructural evolution. While detailed electron back-scattered diffraction (EBSD) quantification of sub-grain size is beyond the scope of this study, fundamental research on similar pearlitic steels has established that gradient deformation significantly refines the interlamellar spacing.^50^^,^^51^ It is postulated that the synergistic effect of lamellar refinement (which maintains geometric hardening) and V-precipitate pinning (which delays dynamic recovery by obstructing dislocation motion) effectively counteracts the dislocation saturation effect. This mechanism sustains the work-hardening capacity even under high deformation levels. Consequently, this “retention of hardening capacity” plays a crucial role in structural safety: a higher and stable *n*-value delays the onset of plastic instability (necking), thereby compensating for the ductility loss typically associated with high-strength steels.

Crucially, Figure 7B reveals that the strain hardening exponent n is not a constant but fluctuates with strain rate. In the classical J-C model, the hardening parameter n is assumed to be independent of strain rate (uncoupled). The observed variation in our experimental data—specifically the rate-dependent and non-monotonic evolution of n-provides empirical evidence that the standard J-C model is insufficient to capture the complex hardening behavior of this vanadium-microalloyed steel. This limitation necessitates the development of a modified constitutive model with coupled strain-rate and hardening terms, capable of describing the evolving *n*-value, which will be rigorously developed and verified in subsequent chapters.

### Fractographic analysis and failure mechanisms

High-carbon pearlitic steels play a critical role in engineering applications, where their fracture behavior is fundamentally governed by the dynamic response of the lamellar ferrite/cementite microstructure. In this investigation, we systematically characterized the tensile fracture morphology of both gradient-rolled vanadium-microalloyed high-carbon steel (steel B) and reference steel (steel A) using scanning electron microscopy (SEM). This comprehensive analysis elucidates the interdependent effects of rolling deformation, strain rate, and vanadium microalloying on fracture mechanisms, revealing their synergistic modulation of material failure behavior.

**Figure 8:**
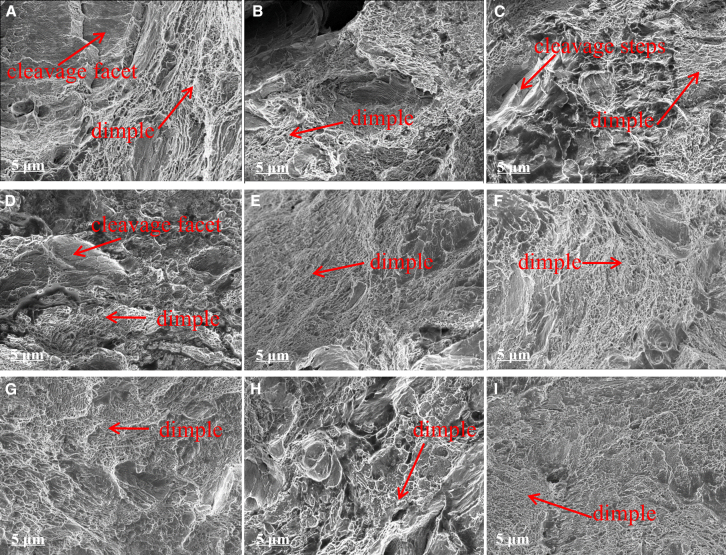
SEM fractographs of steel A tensile specimens under varying conditions (A–C) A-R10, (D–F) A-R20, and (G–I) A-R40 at strain rates of 10^−4^, 10^−2^, and 1.3 s^−1^, respectively (scale bars, 5 μm).

Figure 8 illustrates the evolution of fracture morphologies for the reference steel A under varying rolling reductions and strain rates. The fracture mode is governed by a competitive mechanism between ductile micro-void coalescence and brittle cleavage propagation.

At quasi-static conditions (10^−4^ s^−1^), the specimen with low rolling reduction (A-R10; Figure 8A) exhibits a brittle-dominated fracture characterized by large cleavage facets (∼30 μm) and distinct river patterns, indicating that transgranular cleavage is the primary failure mode. With increasing rolling reduction to 40% (A-R40; Figure 8G), a significant transition occurs: the fracture surface becomes densely populated with fine dimples (∼1 μm), suggesting that severe plastic deformation refines the microstructure and promotes void nucleation, thereby enhancing ductility. This aligns with the increased total elongation observed in mechanical testing.

However, the effect of strain rate reveals a complex transition. Contrary to the behavior at low rates, Intermediate strain rates (1.3 s^−1^) induce a mixed-mode fracture. While Figure 8C shows shallow dimple-like features, these are likely associated with adiabatic shear localization rather than deep plastic flow, given the limited macroscopic ductility. The presence of quasi-cleavage steps in Figure 8F further confirms that high-speed loading suppresses dislocation mobility, limiting the material’s ability to undergo extensive plastic deformation before fracture.

**Figure 9:**
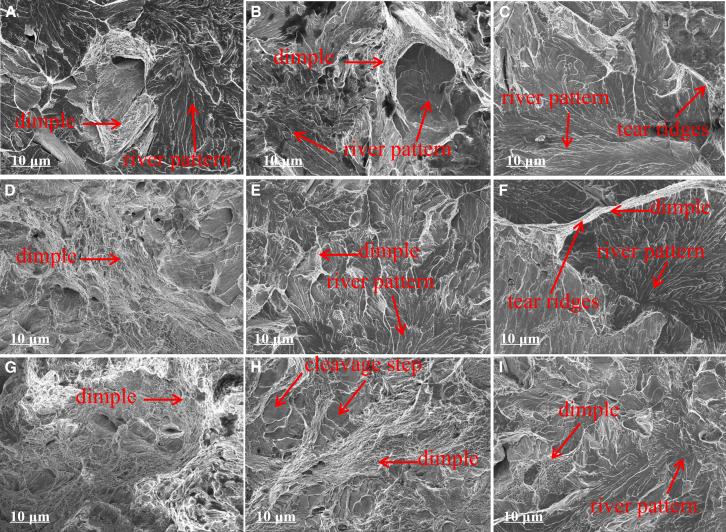
SEM fractographs of steel B tensile specimens under varying conditions (A–C) B-R10, (D–F) B-R20, and (G–I) B-R40 at strain rates of 10^−4^, 10^−2^, and 1.3 s^−1^, respectively (scale bars, 10 μm).

The fracture behavior of the vanadium-microalloyed steel B, presented in Figure 9, exhibits a more pronounced strain-rate sensitivity.

Similar to steel A, increasing rolling reduction significantly suppresses brittle fracture. The B-R40 specimen at low strain rate (Figure 9G) displays a fully ductile morphology with deep, equiaxed dimples, demonstrating that the gradient rolling process effectively toughens the matrix despite the strengthening effect of vanadium.

A distinct ductile-to-brittle transition is observed with increasing strain rate. For B-R10, the fracture surface shifts from a mixed mode at 10^−4^ s^−1^(Figure 9A) to a predominantly brittle mode at 1.3 s^−1^ (Figure 9C). The high-rate fracture surface is characterized by extensive river patterns and tear ridges, indicative of rapid crack propagation along specific crystallographic planes.

**Figure 10:**
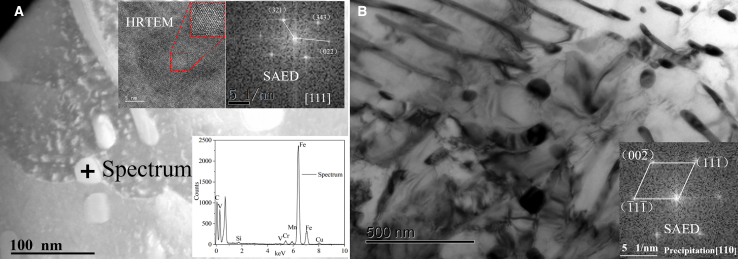
TEM characterization of B-R40 specimen (prior to tensile deformation) (A) The bright field image shows the distribution of nano precipitates, and EDS spectroscopy, SAED, and HRTEM confirm that the precipitates are vanadium carbonitride (inset). (B) The dark field image displays the distribution of nanoscale precipitates and selected area electron diffraction and high-resolution TEM (inset) to identify crystal orientation (scale bars, 100 nm and 500 nm).

To elucidate the micro-mechanism governing this strain-rate-induced embrittlement, TEM characterization was performed on steel B-R40, as shown in Figure 10.

The bright-field images reveal a dispersion of nanoscale precipitates within the ferrite matrix. Energy dispersive spectroscopy (EDS) and selected area electron diffraction (SAED) confirmed that these are elliptical vanadium carbonitrides (V(C, N)). Through the statistical measurement of more than 50 precipitates in multiple dark field images, the average particle size is 50 ± 12 nm. These precipitates play a dual role in the dynamic failure process:

Precipitate pinning effect: the widely dispersed nanoscale V(C,N) precipitates (∼50 nm) naturally act as hard, secondary-phase obstacles to dislocation glide within the softer ferrite matrix. Under quasi-static to low-strain-rate loading, the matrix possesses sufficient time for thermal activation and plastic accommodation around these nano-obstacles. However, under intermediate strain rates (1.3 s^−1^), the time available for thermal activation is severely restricted.

Crack initiation source: the inability of mobile dislocations to effectively bypass these rigid nanoprecipitates at elevated rates leads to severe dislocation pile-ups at the precipitate-matrix interfaces. This results in high local stress concentrations that can readily exceed the cleavage fracture stress of the matrix or the interfacial bonding strength. Consequently, rather than fully toughening the matrix, these precipitates act as competitive initiation sites for the cleavage fractures observed in Figures 9C–9F and 9I. This explicitly explains the competitive failure mechanism where ductile dimple rupture transitions to brittle-dominated quasi-cleavage at higher strain rates.

This observation offers a compelling contrast to recent literature,^11^ where nanoprecipitates were found to activate ultrahigh strength without sacrificing ductility in an ultrafine-grained ferritic matrix during dynamic deformation. While the V(C,N) precipitates in our study similarly provide a remarkable dynamic strengthening effect, the inherently hard lamellar pearlitic matrix, combined with the pre-existing gradient dislocation network, severely restricts local plastic accommodation. Consequently, rather than fully toughening the matrix at elevated strain rates, these precipitates act as competitive cleavage initiation sites, revealing the profound influence of the base microstructure on dynamic precipitation mechanisms.

In summary, while gradient rolling promotes a ductile fracture mechanism through grain refinement, the presence of V-precipitates imposes a kinetic constraint on plasticity under dynamic loading, favoring brittle cleavage fracture.

## Discussion

### Intermediate strain-rate constitutive model

Over recent decades, numerous strain rate-dependent constitutive models have been developed to characterize the dynamic mechanical behavior of rate-sensitive metallic materials. In this study, we employ the widely utilized J-C model to systematically investigate the dynamic constitutive relationships of the studied steels. The J-C model has gained prominence due to its predictive accuracy and mathematical simplicity, making it particularly effective for characterizing the true stress-plastic strain response of metallic materials under varying strain rates.

While previous studies have demonstrated the efficacy of the J-C model in predicting the dynamic behavior of conventional strength steels, its applicability to vanadium-microalloyed high-carbon pearlitic steels remains uncertain. Consequently, a critical evaluation of the J-C model’s suitability for this material system is necessary, along with precise determination of the associated model parameters through comprehensive experimental validation.

### Adiabatic temperature rise and assumption of isothermal deformation

The classical J-C model describes flow stress (*σ*) as a decoupled function of strain hardening, strain rate hardening, and thermal softening:

(Equation 1) $$\sigma =\left(A+B\varepsilon^{n}\right)\left(1+C\;\ln\;{\dot{\varepsilon }}^{*}\right)\left(1-T^{*m}\right)$$

where σ is the plastic stress, *ε* is the plastic strain, ${\dot{\varepsilon }}^{*}$ is the dimensionless plastic strain rate (${\dot{\varepsilon }}^{*}=\dot{\varepsilon }/{\dot{\varepsilon }}_{0}$), and $\dot{\varepsilon }$ is the strain rate, ${\dot{\varepsilon }}_{0}$ usually corresponds to the strain rate of the quasi-static test, and the strain rate of the quasi-static tensile test in this paper is 1 × 10^−4^ s^−1^. *T*∗=(*T*−*T*_*r*_)/(*T*_*m*_−*T*_*r*_), and *T*, *T*_*r*_, and *T*_*m*_ are the test, room, and melting temperatures, respectively. *A* (MPa), *B* (MPa), *C*, *m*, and *n* are the material parameters determined by the experiments.

In classical J-C modeling, the thermal softening term must be considered if elevated loading rates induce significant adiabatic heating. To verify this, the maximum adiabatic temperature rise (Δ*T*) during deformation was estimated by integrating the plastic work from the derived true stress-plastic strain data.^52^^,^^53^ The thermodynamic calculation (detailed in the supplemental information S1) reveals that the estimated temperature increase across all tested strain rates (10^−4^ to 1.3 s^−1^) remains strictly below 30°C (Table 2). Given that high-carbon steels maintain a nearly constant tensile strength below 150°C,^54^ this minimal thermal energy represents only 20% of the threshold for measurable strength reduction and is insufficient to activate significant dislocation recovery mechanisms. Consequently, the deformation process in this study is reasonably treated as isothermal. The thermal softening term is therefore neglected, and the baseline constitutive relationship without temperature effects is simplified as:

(Equation 2) $$\sigma =\left(A+B\varepsilon^{n}\right)\left(1+C\;\ln\;{\dot{\varepsilon }}^{*}\right)$$

**Table 2:** Estimated temperature rise at different strain rates during the stretching process

Strain rate (s^−1^)	1 × 10^−4^	1 × 10^−3^	1 × 10^−2^	1 × 10^−1^	1	1.3
Δ*T* (°C) (A-R10) Δ*T* (°C) (A-R20) Δ*T* (°C) (A-R40) Δ*T* (°C) (B-R10) Δ*T* (°C) (B-R20) Δ*T* (°C) (B-R40)	21 23 23 23 20 16	21 24 23 24 21 17	21 23 23 24 21 20	22 23 23 24 22 20	24 19 22 24 22 22	25 22 24 24 22 24

### Standard J-C model and its limitations

**Figure 11:**
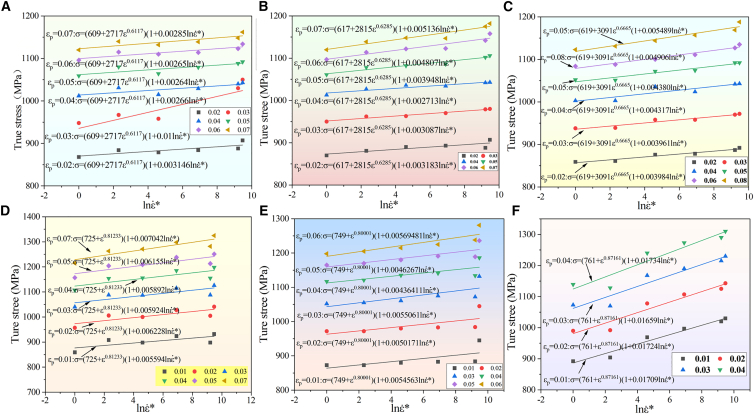
True stress versus dimensionless strain rate (A) A-R10. (B) A-R20. (C) A-R40. (D) B-R10. (E) B-R20. (F) B-R40.

Under quasi-static conditions, ${\dot{\varepsilon }}^{*}$ = 1, (*A* + *Bε*^*n*^) denotes the strain-hardening effect of the true plasticity curve, *A* is defined as the yield stress under quasi-static conditions, and parameters *B* and *n* are the strain-hardening modulus and the hardening index, respectively, which can be determined by fitting the quasi-static curves with logarithmic coordinates. Subsequently, the strain rate sensitivity coefficient (*C*) was derived from the flow stress data at varying strain rates. To maintain conciseness, the detailed mathematical procedures—including the logarithmic transformations and linear regression steps used for this standard calibration—have been compiled in the supplemental information (Text S2). The obtained baseline parameters are summarized in Table 3, and the linear fits for parameter *C* are displayed in Figure 11.

**Table 3:** Material parameters of J-C model

Parameters	*A* (MPa)	*B* (MPa)	*C*	*n*
A-R10–1×10^−4^ s^−1^	609	2,717	0.003980	0.6117
A-R20–1×10^−4^ s^−1^	617	2,835	0.003812	0.6284
A-R40–1×10^−4^ s^−1^	619	3,091	0.004506	0.6665
B-R10–1×10^−4^ s^−1^	725	6,056	0.01986	0.8716
B-R20–1×10^−4^ s^−1^	749	4,063	0.005111	0.8000
B-R40–1×10^−4^ s^−1^	815	4,328	0.002838	0.8123

As shown in Table 3, the strain rate sensitivity coefficient (*C*) decreases significantly from 0.01986 (B-R10) to 0.002838 (B-R40). This phenomenon is attributed to the saturation of dislocation density induced by severe gradient rolling. In the B-R40 specimen, the pre-existing high density of dislocations and the pinning effect of V-precipitates significantly reduce the effective thermal activation volume for dislocation motion. Consequently, the flow stress becomes less sensitive to strain rate variations compared to the lightly deformed B-R10 specimen, where thermally activated dislocation glide is more pronounced.

In order to verify the applicability of the standard J-C model for A and B steels at different levels of rolling, Figures 14 and 15 show the experimental data for A-R40 and B-R40 compared with the predicted curves from the standard J-C model. The models agree well with the experimental data at low strain rates, but show significant deviations at higher strain rates (1.3 s^−1^), especially in the high plastic strain region. For vanadium microalloyed high-carbon steels, the interaction between VC precipitated phases and dislocations is exacerbated at Intermediate strain rates, resulting in an underestimation of the flow stresses by the model (the experimental curves are higher than the predicted curves at high strain rates in Figures 14 and 15).

### Modified J-C model with cubic polynomial coupling

To accurately capture the multi-stage work hardening characteristics, a modified J-C model is proposed. The standard power-law term is replaced by a cubic polynomial function, coupled with the strain rate effect:

(Equation 3) $$\sigma =\left(A+B_{1}\varepsilon +B_{2}\varepsilon^{2}+B_{3}\varepsilon^{3}\right)\left(1+C\;\ln\;{\dot{\varepsilon }}^{*}\right)$$

where *σ* is the macroscopic flow stress (MPa); *ε* is the true plastic strain; and ${\dot{\varepsilon }}^{*}$ is the dimensionless equivalent plastic strain rate. The specific definitions, units, and physical meanings of the model parameters are detailed as follows:

*A* (MPa): the yield strength under quasi-static conditions, representing the initial deformation resistance of the material.

*B*_1_*ε* (MPa): the first-order (linear) coefficient, which dominates the hardening rate at the initial stage of strain hardening (corresponding to the rapid dislocation multiplication phase).

*B*_2_*ε* (MPa): the second-order (quadratic) coefficient. It is typically a negative value, characterizing the suppressive effect of dynamic recovery or dislocation annihilation on the overall hardening rate.

*B*_3_*ε* (MPa): the third-order (cubic) coefficient, describing the secondary hardening or hardening saturation behavior at large strains. Physically, it is associated with the evolution of long-range interactions between dislocations and microstructural barriers, such as precipitates and grain boundaries.

*C* (dimensionless): the strain rate sensitivity coefficient, reflecting the rate at which the material’s flow stress increases with the logarithmic increase of the applied strain rate.

**Figure 12:**
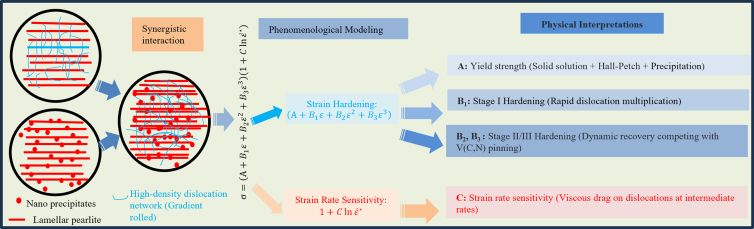
Schematic diagram of the proposed modified J-C constitutive modeling framework The flowchart bridges the experimental microstructural features (gradient dislocation network and V-precipitates) with the macroscopic mathematical formulation, explicitly mapping the phenomenological parameters (*A*, *B*_1_, *B*_2_, *B*_3_, and *C*) to their underlying physical interpretations.

To facilitate a clear understanding of the proposed modeling framework and its physical significance, a schematic diagram mapping the phenomenological mathematical parameters to their corresponding metallurgical mechanisms is presented in Figure 12. As illustrated, the constitutive formulation bridges the gap between the macroscopic dynamic response and the underlying gradient-rolled microstructure. The synergistic interaction between the pre-existing high-density dislocation network and the nanoscale V(C,N) precipitates directly dictates the evolution of the hardening coefficients (*B*_1_, *B*_2_, *B*_3_) and the strain rate sensitivity parameter (*C*).

### Parameter identification and verification

**Figure 13:**
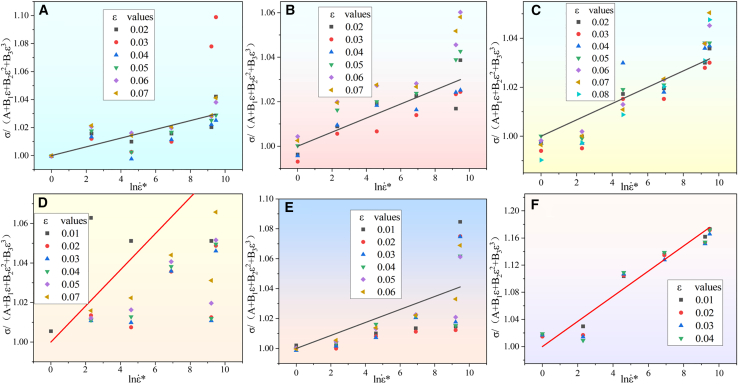
Relationship between *σ*/(*A* + *B*_1_*ε*+*B*_2_*ε*^2^+*B*_3_*ε*^3^)and $\ln\;{\dot{\varepsilon }}^{*}$ (A) A-R10, (B) A-R20, (C) A-R40, (D) B-R10, (E) B-R20, and (F) B-R40.

The model parameters were calibrated using a two-step regression method. First, the quasi-static curves were fitted to obtain *A*, *B*_1_, *B*_2_, and *B*_3_. Subsequently, the strain rate sensitivity parameter *C*was determined by plotting the normalized stress against $\ln\;{\dot{\varepsilon }}^{*}$, as shown in Figure 13. The calibrated parameters are summarized in Table 4.

**Table 4:** Material parameters for the modified J-C model

Parameters	*A* (MPa)	*B*_1_	*B*_2_	*B*_3_	*C*
A-R10–1×10^−4^ s^−1^	609	16,624	−195,460	877,167	0.00312
A-R20–1×10^−4^ s^−1^	617	16,334	−193,933	895,226	0.00316
A-R40–1×10^−4^ s^−1^	619	15,088	−162,713	682,209	0.00334
B-R10–1×10^−4^ s^−1^	725	14,229	−134,416	449,364	0.00915
B-R20–1×10^−4^ s^−1^	749	13,358	−116,419	310,358	0.00434
B-R40–1×10^−4^ s^−1^	761	12,704	−103,142	215,507	0.01854

**Figure 14:**
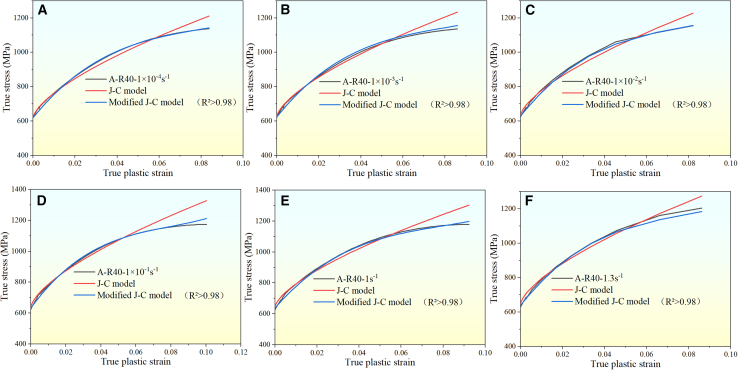
Comparison of experimental results and models of A-R40 at various strain rates (A) 10^−4^ s^−1^, (B) 10^−3^ s^−1^, (C) 10^−2^ s^−1^, (D) 10^−1^ s^−1^, (E) 1 s^−1^, and (F) 1.3 s^−1^.

**Figure 15:**
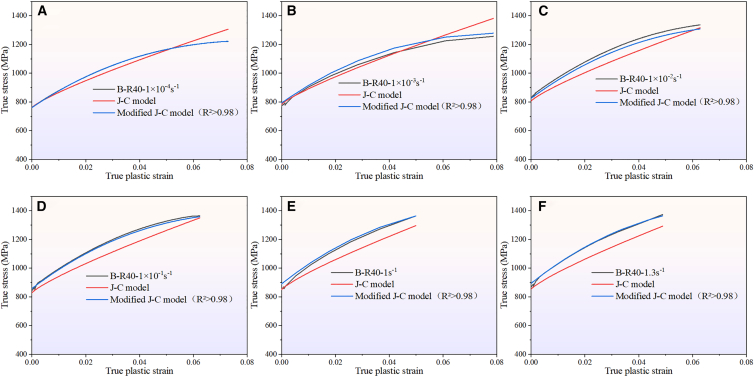
Comparison of experimental results and models of B-R40 at various strain rates (A) 10^−4^ s^−1^, (B) 10^−3^ s^−1^, (C) 10^−2^ s^−1^, (D) 10^−1^ s^−1^, (E) 10^−1^ s^−1^, and (F) 1.3 s^−1^.

Figures 14 and 15 compare the experimental true stress-strain curves with the predicted results of the modified constitutive model for the representative A-R40 and B-R40 specimens at various strain rates. It is evident that the proposed model accurately captures the dynamic plastic flow behavior, including the initial yielding and the subsequent non-linear strain hardening. To quantitatively evaluate the predictive accuracy across all process conditions, the correlation coefficients (R^2^) were calculated. The modified model demonstrates superior predictive accuracy, achieving values consistently exceeding 0.98 for all tested specimens. For conciseness, the comparison curves for the remaining specimens (A-R10, A-R20, B-R10, and B-R20) and the detailed values are provided in Figures S2–S5 and Table S2 in the supplemental material. This substantial improvement confirms that the cubic polynomial coupling term effectively captures the synergistic effects of gradient rolling and vanadium precipitation on the dynamic flow stress.

### Physical interpretation and microstructure-parameter correlation

As conceptually illustrated in Figure 12, to elevate the proposed constitutive model from purely empirical to physically informed, a correlation analysis between the calibrated model parameters (*A*, *B*_*i*_, *C*), process conditions, and microstructural features was conducted. The evolution of these parameters reveals the underlying mechanisms governing the macroscopic constitutive behavior.

#### Quasi-static yield parameter (*A*)

The dominant static factor, parameter *A*, physically represents the initial resistance to plastic flow. Its magnitude is fundamentally dictated by the measurable microstructural features. Specifically, the quantitatively measured refinement in the average pearlite interlamellar spacing (*λ*) discussed earlier (e.g., refining down to 123 nm in B-R40) provides a robust geometric basis for the monotonic increase in parameter *A*. In steel B, this baseline is further elevated by the uniform dispersion of the quantitatively confirmed nanoscale V(C,N) precipitates (∼50 nm average diameter).

#### Evolution of hardening coefficients ($B_{i}$)

The polynomial coefficients *B*_1_, *B*_2_, and *B*_3_describe the multi-stage hardening process. Their absolute values generally decrease with increasing rolling reduction, directly reflecting the evolution of internal defect structures.

*B*_1_ represents the initial hardening rate. Gradient rolling introduces a high pre-existing dislocation density. According to the Kocks-Mecking theory, the dislocation proliferation rate is inversely proportional to the current dislocation density. Thus, highly deformed specimens (e.g., B-R40) exhibit a significantly lower *B*_1_ compared to lightly deformed ones, indicating that the capacity for rapid dislocation multiplication has been pre-exhausted by the gradient rolling process.

*B*_2_ and *B*_3_ govern the dynamic recovery and saturation behavior at large strains (secondary hardening). Notably, the *B*_3_ values for steel B are significantly lower than those for steel A. This is physically correlated to the strong pinning effect of nanoscale V(C,N) precipitates and the refined pearlite interlamellar spacing. While these microstructural features increase the yield strength (*A*), they severely restrict the dislocation mean free path, thereby accelerating dynamic recovery (*B*_2_) and suppressing the secondary hardening capability (*B*_3_) at large plastic strains. This explains the flatter terminal slope of the stress-strain curves for the vanadium-alloyed steel.

#### Strain rate sensitivity (*C*)

Based on the modified model (Table 4), the strain rate sensitivity coefficient *C* increases with gradient rolling reduction, rising from 0.0092 (B-R10) to 0.0185 (B-R40). This parameter is a macroscopic reflection of the effective thermal activation volume. In the B-R40 specimen, the ultra-high density of dislocation forests entangles with nanoscale V(C,N) precipitates. Under intermediate strain rate loading, the time available for thermal activation is insufficient for mobile dislocations to bypass these dense microstructural obstacles. Consequently, the flow stress exhibits a steeper response to strain rate increments (higher *C* value) compared to the lightly deformed state. This confirms that the synergistic effect of precipitation and dislocation forest hardening makes the material’s strength inherently more sensitive to loading rates.

#### Parameter correlation

A distinct negative correlation is observed between the third-order hardening coefficient *B*_3_ and the strain rate sensitivity *C*, bridging microstructural features with macroscopic responses. High *B*_3_ values (typically found in the reference steel A) correspond to low *C* values, indicating a deformation mechanism dominated by long-range dislocation interactions within the softer lamellar matrix. Conversely, the combination of low *B*_3_ and high *C* values in the highly deformed steel B (B-R40) suggests a regime dominated by short-range precipitate pinning. The high *C* value physically represents the increased viscous drag on dislocations imposed by V(C,N) precipitates, while the low *B*_3_ reflects the early saturation of work hardening. This trade-off correlation establishes a robust physical universality for modeling the material’s response under intermediate strain rate loading.

#### Sensitivity analysis

To evaluate the robustness of the proposed model, a single-parameter sensitivity analysis was conducted. Varying the yield parameter *A* by ±10% results in a proportional shift in flow stress, confirming it as the dominant static factor. In contrast, varying the strain rate sensitivity *C* by ±10% has a negligible effect at quasi-static rates but alters the dynamic flow stress by approximately 3%–5% at 1.3 s^−1^ (due to the higher calibrated *C* value in the modified model). The polynomial terms (*B*_1_, *B*_2_, *B*_3_) show strain-dependent sensitivity, validating their specific roles in capturing the non-linear work-hardening evolution across different deformation stages.

### Adaptability to actual service conditions and complex stress states

To apply the proposed one-dimensional constitutive model to actual service conditions, it must be generalized to accommodate complex stress states. In practical engineering applications, such as seismic excitations, wind-induced vibrations, or vehicle impacts on bridge structures, components are rarely subjected to simple uniaxial loading; instead, they experience multiaxial stress states.

The proposed model can be implemented into a three-dimensional FE framework using an equivalent stress-strain approach, such as the von Mises yield criterion and the associated flow rule. The accurately calibrated strain rate sensitivity and multi-stage hardening parameters are critical for predicting the dynamic plastic flow and energy absorption of the gradient-rolled steel under multiaxial loading. Furthermore, the gradient microstructure itself—featuring a high-strength surface and a ductile core—exhibits excellent adaptability to complex stress states by delaying strain localization and preventing catastrophic crack propagation.

However, it should be noted that damage initiation and fracture in actual service are highly sensitive to the stress state, specifically the stress triaxiality and Lode angle parameter. While the current model successfully captures the dynamic plastic flow behavior across different strain rates, coupling the proposed constitutive equations with a stress-state-dependent damage criterion (e.g., the modified Mohr-Coulomb or Bai-Wierzbicki fracture model) will be a necessary next step to fully predict the failure limits of these gradient-structured steels under extreme complex service conditions.

### Comprehensive evaluation of the proposed model

To objectively evaluate the proposed modified J-C model, a broader contextual discussion and a side-by-side comparison with the standard J-C model and alternative forms are necessary.

#### Qualitative and quantitative comparison

Qualitatively, the standard J-C model relies on a monotonic power-law function (*Bε*^*n*^) for strain hardening. While effective for homogeneous materials, it fundamentally fails to capture the inflection points and early saturation of work hardening caused by the dense V(C,N) precipitates and gradient dislocation forests in the current microalloyed steel. In contrast, the proposed model replaces the power-law term with a cubic polynomial function (*B*_1_*ε*+*B*_2_*ε*^2^+*B*_3_*ε*^3^). This mathematical flexibility accurately traces the multi-stage hardening trajectory—from rapid initial dislocation multiplication to dynamic recovery and precipitate pinning. Quantitatively, fitting the standard J-C model to our highly deformed datasets typically yields noticeable deviations at large strains. By implementing the polynomial modification, the quantitative predictive accuracy is significantly boosted, achieving values consistently exceeding 0.98 across all tested conditions.

#### Advantages

Compared to complex physically based models (e.g., dislocation-density evolution models or the Zerilli-Armstrong model), the primary advantage of the proposed model lies in its decoupled phenomenological framework. It successfully bridges microstructural mechanisms (via parameter physicalization, as discussed in earlier in the physical interpretation of model parameters) with macroscopic mathematical simplicity. This makes the model highly efficient and straightforward to implement into commercial three-dimensional FE software (e.g., via VUMAT in Abaqus) for large-scale engineering simulations.

#### Expected applicability range

As defined by the experimental calibration, the expected applicability range of the proposed formulation is strictly bounded within the quasi-static to intermediate strain rate regime (10^–4^ to 1.3 s^−1^) at room temperature. This range accurately represents typical loading conditions for bridge cables, such as wind-induced vibrations and seismic excitations, making the model highly applicable for structural safety assessments.

This study systematically investigated the dynamic tensile behavior and constitutive modeling of vanadium-microalloyed high-carbon pearlitic steel processed by gradient rolling (10%–40% reduction) under strain rates ranging from 10^−4^ to 1.3 s^−1^. By integrating macro-mechanical testing with microstructural characterization (SEM/TEM), the following key conclusions are drawn regarding the material’s suitability for long-span bridge cables.

(1) The combination of vanadium microalloying and gradient rolling yields a superior strength-ductility balance. Steel B (V-alloyed) exhibits a yield strength exceeding 700 MPa and an ultimate tensile strength over 1,299 MPa at intermediate strain rates, outperforming the reference steel A by ∼25%. Although intermediate strain rate and large rolling reductions induce a trade-off in ductility, the material maintains a total elongation greater than 6% even under the most severe loading conditions (1.3 s^−1^), providing sufficient safety margins against catastrophic failure in seismic events.
(2) Quantitative analysis of the Hollomon parameters reveals a counter-intuitive hardening behavior. Unlike typical cold-worked metals, the strain hardening exponent (*n*) of the V-microalloyed steel does not degrade with increasing rolling reduction but remains stable or slightly increases (from 0.24 to 0.26). This “retention of hardening capacity” is attributed to the refinement of pearlite nodules and the effective interaction between dislocations and V-precipitates, which delays the onset of plastic instability (necking).
(3) Fractography reveals a competitive mechanism governing dynamic failure. While gradient rolling promotes ductile dimple fracture through grain refinement, elevated strain rates trigger a ductile-to-brittle transition. TEM analysis confirms the homogenous dispersion of nanoscale V(C,N) precipitates (∼50 nm). Instead of asserting specific dislocation bypass mechanisms, we objectively conclude that at elevated strain rates, these rigid nanoprecipitates act as strong kinetic obstacles. The restricted thermal activation volume leads to severe interfacial dislocation pile-ups and localized stress concentrations, transforming these precipitates into cleavage initiation sites. This governs the emergence of quasi-cleavage features and river patterns observed at 1.3 s^−1^.
(4) The standard J-C model proved insufficient to describe the non-linear hardening behavior and the rate-dependent evolution of the *n*-value. Consequently, a modified Johnson-Cook model incorporating a cubic polynomial coupling term was developed. This physical-based modification successfully captures the multi-stage hardening process (initial hardening, dynamic recovery, and saturation). Compared to the standard formulation, the proposed model significantly enhances the prediction accuracy, achieving a coefficient of determination (R^2^) exceeding 0.98. The proposed model offers a robust theoretical tool for the nonlinear dynamic analysis and structural integrity assessment of next-generation bridge cables under intermediate strain rates (10^−4^ to 1.3 s^−1^), such as wind-induced vibrations and seismic excitations. Future research will focus on high-strain-rate experiments (e.g., Split-Hopkinson Pressure Bar testing) to recalibrate thermal softening effects and broaden the model’s universality for extreme impact safety assessments.

### Limitations of the study

Despite its advantages, the current model exhibits specific limitations that outline future research directions. First, extrapolation to extremely high strain rates (>10^2^ s^−1^), associated with blast or ballistic impacts, should be undertaken with caution. Under such extreme conditions, the isothermal assumption may no longer hold due to significant adiabatic heating. Future studies employing split-Hopkinson pressure bar (SHPB) testing will be necessary to re-calibrate the thermal softening terms. Second, as an inherently phenomenological continuum model, the modified J-C formulation captures microstructural heterogeneity implicitly. To explicitly incorporate the spatial distribution of grain sizes, anisotropic thermal stresses, and localized shear banding under extreme conditions, future work must transition from this macroscopic formulation to a multi-scale crystal plasticity FE method (CPFEM) framework. Integrating the current dynamic laws into a CPFEM model will enable the explicit resolution of thermo-mechanical coupling at the microstructural level.

## Resource availability

### Lead contact

Further information and requests for resources and materials should be directed to and will be fulfilled by the lead contact, Changrong Li (crli@gzu.edu.cn).

### Materials availability

This study did not generate new unique reagents.

### Data and code availability

• All data reported in this paper will be shared by the lead contact upon request.
• This paper does not report original code.
• Any additional information required to reanalyze the data reported in this paper is available from the lead contact upon request.

## Acknowledgments

This work was supported by 10.13039/501100001809National Natural Science Foundation of China (grant no. 52464037). Supported by Guizhou Provincial Program on Commercialization of Scientific and Technological Achievements Development and Application of Key Technology of Deep Drawn High Carbon Steel Coil for Tire Rim and Cord Wire and Special Wire Products (grant no. QKHCG [2023]YB100).

## Author contributions

S.H., writing – review and editing, writing – original draft, visualization, project administration, methodology, investigation, formal analysis, data curation, and conceptualization; Z.Y.L., writing – review and editing, validation, software, investigation, formal analysis, and data curation; H.Y., visualization, validation, supervision, and project administration; Z.Y.Z., software, resources, and investigation; Y.J.S., software, validation, and data curation; C.R.L., writing – review and editing, writing – original draft, methodology, investigation, validation, resources, and funding acquisition.

## Declaration of interests

The authors declare no competing interests.

## STAR★Methods

### Key resources table

**Table:** 

REAGENT or RESOURCE	SOURCE	IDENTIFIER
**Software and algorithms**		

OriginPro2023	OriginLab	https://www.originlab.com/
Instron 5982 universal testing system	Instron	https://www.instron.com/
ZEISS Sigma 300 Scanning Electron Microscope	Carl Zeiss	https://www.zeiss.com/
FEI Talos F200x Transmission Electron Microscope	Thermo Fisher Scientific	https://www.thermofisher.com/

### Method details

#### Material preparation and processing

The experimental materials were produced using a 50 kg vacuum induction furnace to ensure high purity (oxygen content <50 ppm). The ingots, with dimensions of 120 mm × 340 mm, were homogenized at 1200°C for 10 min and subsequently subjected to multi-directional forging between 850°C and 1150°C to eliminate casting defects, resulting in a final thickness of 30 mm. The chemical compositions of the reference steel (Steel A) and the vanadium-microalloyed steel (Steel B) are listed in Table 1.

To optimize the dissolution of vanadium in the austenite matrix, the forged plates underwent a specific thermal cycle: austenitization at 1200°C for 30 min, followed by air cooling (average rate of 20 °C/s, estimated from continuous temperature recordings using an infrared pyrometer). Rectangular billets (100 mm × 100 mm×30 mm) were then machined and reheated to 1200°C for 30 min to ensure microstructural homogenization. Prior to rolling, the billets were cooled to 950°C and held for 10 min to stabilize the temperature.

Gradient rolling deformation was simulated by applying varying total reduction levels using a Φ450 mm two-high reversible rolling mill with a linear velocity of 1.5 m/s. The rolling was conducted along the longitudinal direction with a consistent single-pass reduction of 3 mm. To achieve the target total reductions of 10%, 20%, and 40%, the billets underwent 1 pass, 2 passes, and 4 passes, respectively. The inter-pass time was strictly controlled within 5 s to minimize static recrystallization of austenite. Immediately after the final pass, the plates were air-cooled to 550 °C, followed by isothermal aging for 30 min to promote pearlite transformation and vanadium precipitation, and finally air-cooled to room temperature. The entire sample processing flowchart is shown in Figure 1A. The processed specimens are designated as X-Rε, where X denotes the steel grade (A or B) and ε represents the rolling reduction percentage (e.g., B-R40 refers to Steel B with 40% reduction).

#### Intermediate strain-rate mechanical testing and characterization

Uniaxial tensile tests were conducted at room temperature using an Instron 5982 universal testing system, equipped with a 100 kN dynamic load cell (5 kHz frequency response) to capture transient stress waves. To accurately measure strain under dynamic conditions, a high-precision laser extensometer (accuracy ±0.5 μm) was employed, monitoring the real-time deformation within the gauge length. Dog-bone shaped specimens were designed and machined in accordance with ISO 6892-1:2016 standards to minimize geometric effects under dynamic loading.^55^

As illustrated in Figure 1, the specimen geometry features a parallel gauge length of 15 mm, a gauge width of 4 mm, a thickness of 1 mm, and a fillet radius of 4 mm at the transition zone to prevent stress concentration outside the gauge section. To ensure data consistency and evaluate the mechanical response along the principal deformation axis, all specimens were extracted parallel to the rolling direction (RD). Furthermore, to minimize the influence of surface effects and represent the bulk material properties, the specimens were sampled from the mid-thickness position of the rolled plates.

To systematically investigate the strain rate sensitivity, tests were performed across a wide range of engineering strain rates: 10^−4^ s^−1^(quasi-static), 10^−3^ s^−1^, 10^−2^ s^−1^, 10^−1^ s^−1^, 1 s^−1^, and 1.3 s^−1^. The maximum intermediate strain rate of 1.3 s^−1^was specifically selected based on the upper operational limit of the Instron 5982 universal testing system. The nominal strain rate ($\dot{\varepsilon }$) was controlled by adjusting the crosshead velocity (v) according to the relationship $\dot{\varepsilon }=v/L_{0}$, where *L*_0_ is the initial gauge length. Three replicates were tested for each condition to ensure statistical reliability, and the representative curve closest to the average response was selected for analysis.

#### Microstructural analysis

Fractographic features of the tested specimens were examined using a ZEISS Sigma 300 Scanning Electron Microscope (SEM) to elucidate the failure mechanisms. For detailed microstructural characterization, Transmission Electron Microscope (TEM) foils were sliced from the mid-thickness of the rolled plates, parallel to the rolling direction (RD-ND plane), to accurately observe the deformed substructures. The discs (Φ3 mm) were mechanically ground to a thickness of ∼50 μm and subsequently thinned using a twin-jet electropolisher. The electropolishing was performed in a solution of 10% perchloric acid and 90% ethanol at −20 °C, with an applied voltage of 20 V and a current of approximately 40–60 mA, until perforation occurred. The precipitation behavior and dislocation substructures were analyzed using an FEI Talos F200x Transmission Electron Microscope (TEM) operated at 200 KV, equipped with an Energy Dispersive Spectrometer (EDS) for elemental mapping.

### Quantification and statistical analysis

To ensure statistical reliability, three independent tensile tests were performed for each condition. The “average response curve” was mathematically generated by calculating the arithmetic mean of the stress values at corresponding strain increments across the three tests. The specific experimental trial that exhibited the minimum sum of squared errors relative to this calculated average was selected as the representative curve for subsequent constitutive modeling. The interlamellar spacing of pearlite was statistically evaluated via high-magnification SEM imaging, with over 100 measurements per condition using the mean linear intercept method.
