# Supplementary material for: Intermediate strain rate constitutive modeling of gradient rolled vanadium-microalloyed pearlitic steel for bridge cables
Source: iScience. 2026 Jun 10;29(6):116319. doi: 10.1016/j.isci.2026.116319 (PMC13267574; doi:10.1016/j.isci.2026.116319)
Supplement: Document S1. Figures S1–S5, Tables S1 and S2, and Data S1 and S2 [file mmc1.pdf]

iScience, Volume *29*

Supplemental information

Intermediate strain rate constitutive modeling of gradient rolled vanadium-microalloyed pearlitic steel for bridge cables

Sheng Huang, Zhiying Li, Hui Yang, Zeyun Zeng, Yingjie Shi, and Changrong Li

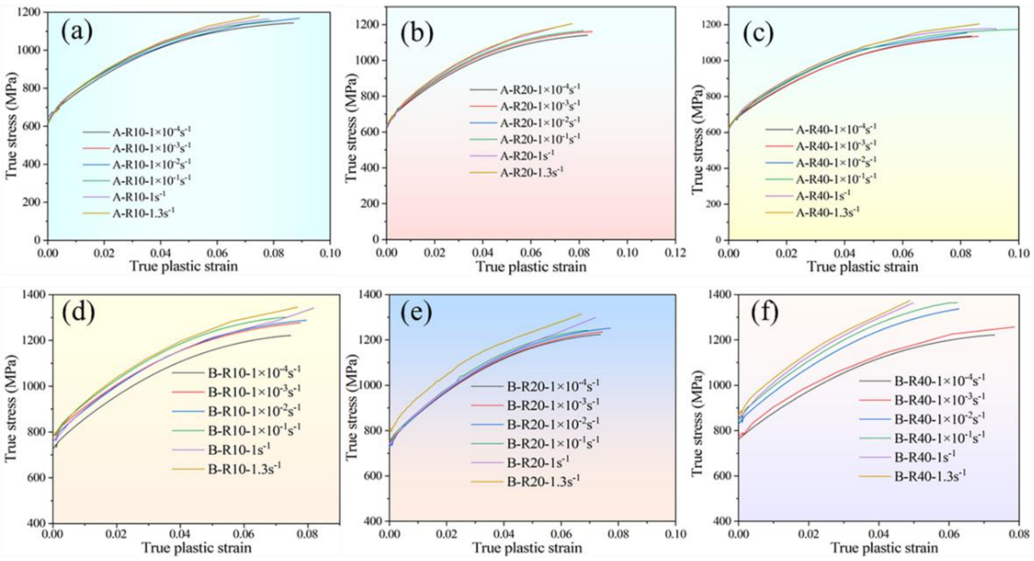

Figure S1. True stress-plastic strain curves: (a) A-R10; (b) A-R20; (c) A-R40; (d) B-R10; (e) B-R20; (f) B-R40

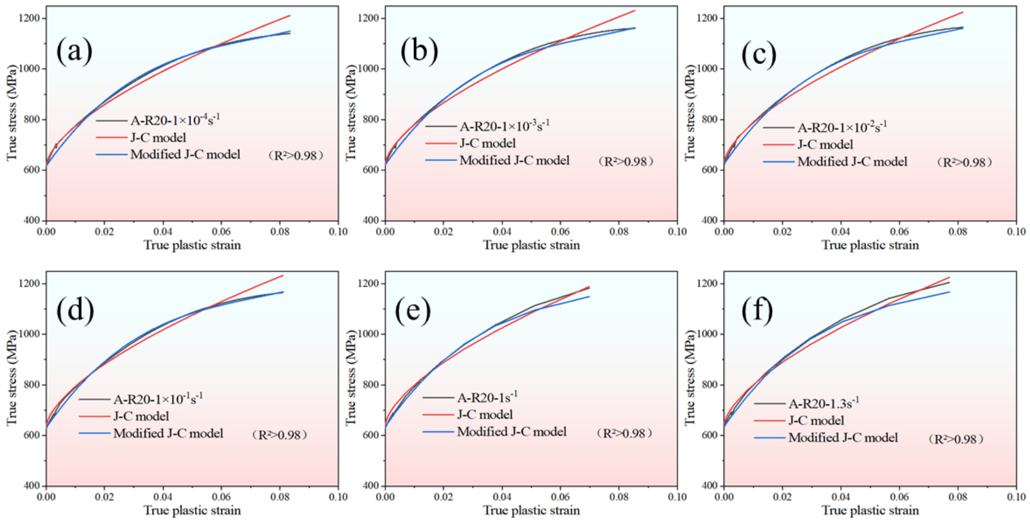

Figure S2. Comparison of experimental results and models of A-R20 at various strain rates

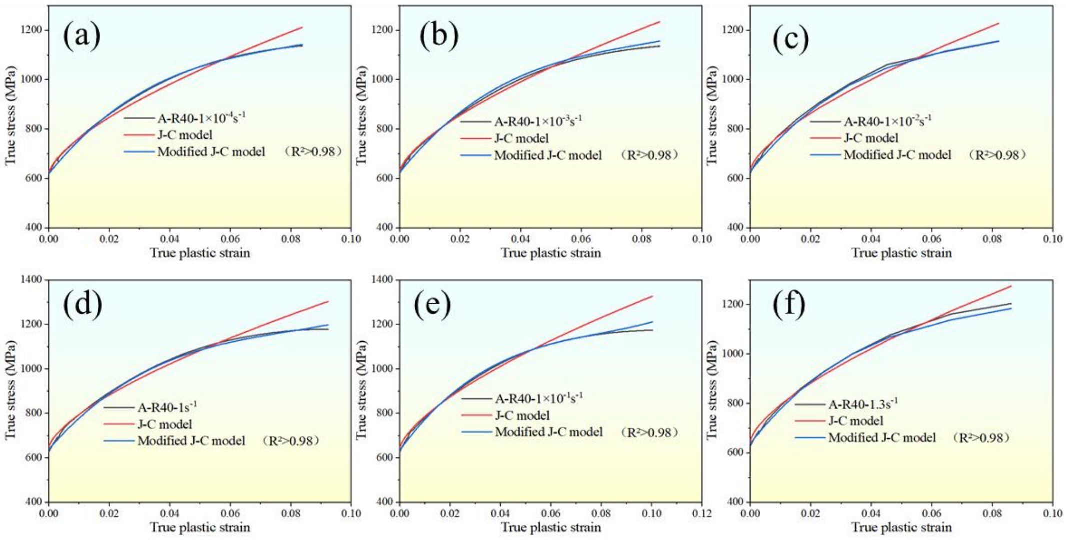

Figure S3. Comparison of experimental results and models of A-R40 at various strain rates

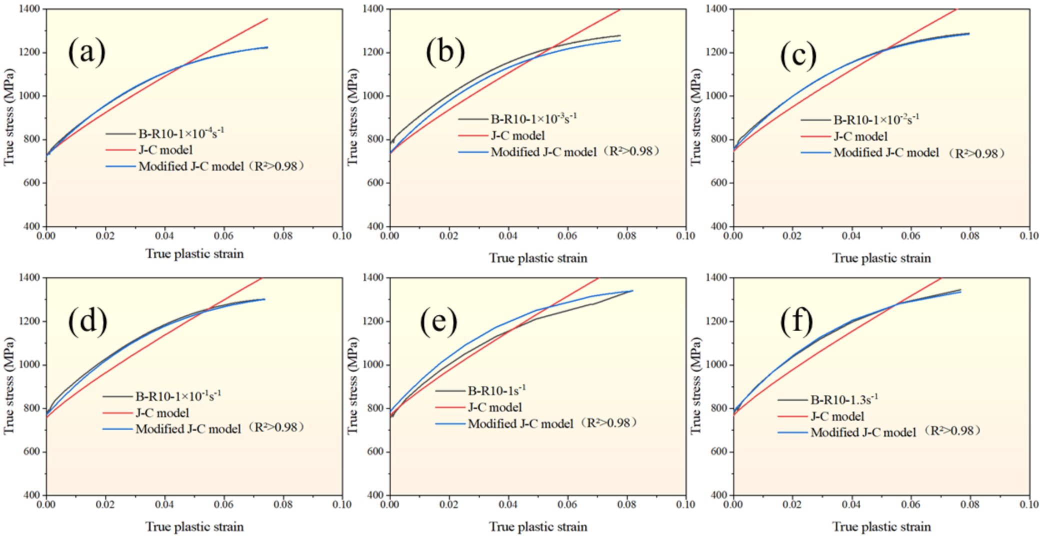

Figure S4. Comparison of experimental results and models of B-R10 at various strain rates

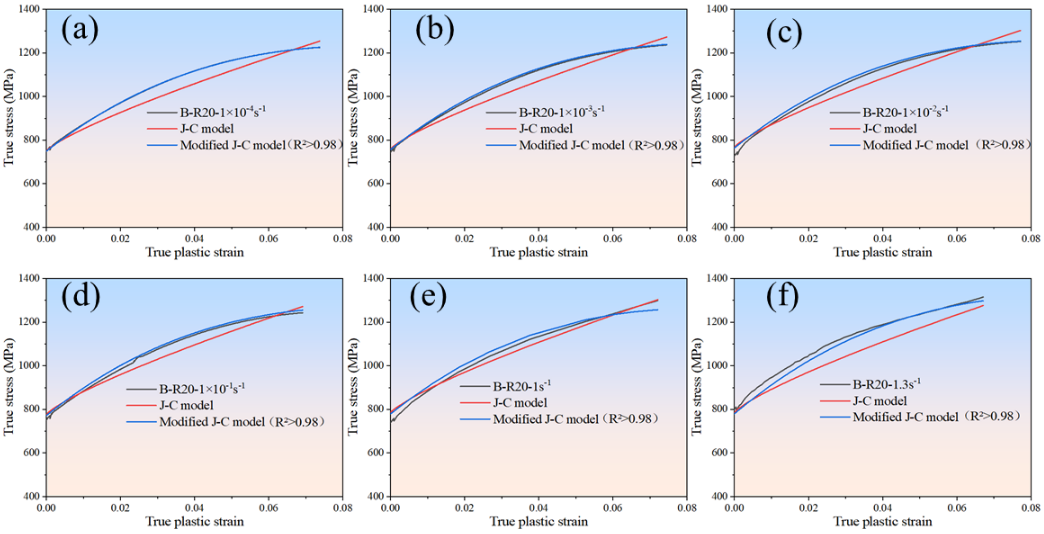

Figure S5. Comparison of experimental results and models of B-R20 at various strain rates

Table S1. Mechanical property information of steel at different strain rates

Steel	Strain rate (s^{-1})	Ultimate strength (MPa)	Yield strength (MPa)	Uniform elongation (%)	Total elongation (%)
	0.0001	1044	609	9.5	11.1
	0.001	1060	612	8.4	10.1
A-R10	0.01	1061	618	8.6	12
	0.1	1062	620	8.6	10.1
	1	1071	626	8.3	10.1
	1.3	1090	644	8.2	10.1
	0.0001	1045	617	8.6	10.3
	0.001	1062	620	8.6	9.7
A-R20	0.01	1069	622	9.2	9.4
	0.1	1071	627	9.1	9.6
	1	1097	629	7.8	7.9
	1.3	1110	637	8.6	10.7

	0.0001	1040	619	10.7	12.6
	0.001	1039	621	10.6	12.3
A-R40	0.01	1058	623	11.1	12.3
	0.1	1063	625	10.4	13
	1	1076	626	9.1	10.4
	1.3	1099	628	9.5	10.7
	0.0001	1126	725	8.8	9.1
	0.001	1174	780	8.8	10.5
B-R10	0.01	1180	755	9.0	11.4
	0.1	1200	777	8.4	10.0
	1	1227	756	9.2	9.8
	1.3	1238	785	8.6	9.5
	0.0001	1130	749	8.4	9.3
	0.001	1139	744	8.5	10.1
B-R20	0.01	1150	728	8.7	9.1
	0.1	1152	752	7.9	9.1
	1	1200	740	8.2	8.9
	1.3	1220	799	7.7	8.2
	0.0001	1150	761	8.5	10.7
	0.001	1154	772	8.9	8.9
B-R40	0.01	1247	828	7.2	7.3
	0.1	1272	844	7.2	7.3
	1	1288	851	5.8	6.7
	1.3	1299	867	5.7	6.3

Table S2. Correlation coefficients (R^2) of the modified J-C model across all tested conditions.

Strain rate (s^{-1})	A-R10	A-R20	A-R40	B-R10	B-R20	B-R40
0.0001	0.998	0.997	0.997	0.998	0.998	0.998
0.001	0.994	0.993	0.993	0.983	0.996	0.982
0.01	0.995	0.998	0.996	0.996	0.995	0.983
0.1	0.988	0.987	0.989	0.989	0.988	0.994
1	0.987	0.985	0.986	0.981	0.983	0.989
1.3	0.983	0.981	0.985	0.989	0.981	0.998

Date S1. Calculation of adiabatic temperature rise

To characterize adiabatic temperature evolution, the temperature increase (ΔT) is calculated as:

$$\Delta \mathrm{T} = \frac{\eta}{\rho C_p} \int \sigma d\varepsilon \quad (1)$$

where ΔT refers to the increase in temperature, η is a measure of how much energy is converted to heat (assumed to be 1 in this paper), ρ is the density of steel, and Cp is the specific heat capacity (ρ=7810 kg/$m^3$and Cp=466 J/(kg·°C). Based on the experimental true stress-plastic strain curves, the estimated ΔT across all strain rates (10^{-4} to 1.3 s^{-1}) remains below 30 ℃. Since this is significantly below the 150 ℃threshold for measurable thermal softening in high-carbon pearlitic steels, the deformation process is treated as isothermal, and

the thermal softening term in the Johnson-Cook model is neglected, simplifying the constitutive model to:

$$\sigma = (A + B\varepsilon^n)(1 + C \ln \dot{\varepsilon}^*) \qquad (2)$$

Date S2. Standard Johnson-Cook model and its limitations

Under quasi-static conditions, $\dot{\varepsilon}^*$=1，$(A + B\varepsilon^n)$ denotes the strain-hardening effect of the true plasticity curve, A is defined as the yield stress under quasi-static conditions, and parameters B and n are the strain-hardening modulus and the hardening index, respectively, which can be determined by fitting the quasi-static curves with logarithmic coordinates. The formula is: ln(σ-A) = ln(B) + nln(ε), and the parameters B and n are obtained by slope and intercept, and the results are shown in Table 3 of the main manuscript.

C is a fundamental parameter characterizing the strain rate effect, and based on the experimental results of the true stress-plastic strain relationship (e.g., Fig. S1), the value of C is fitted by Equation:

$$\sigma = \sigma_s + \sigma_s \cdot C ln\dot{\varepsilon}^* \quad (3)$$

Where $\sigma_s = A + B\varepsilon^n$. The obtained baseline parameters are provided in Table 3 of the main manuscript.
